# Features of floral odor and nectar in the distylous *Luculia pinceana* (Rubiaceae) promote compatible pollination by hawkmoths

**DOI:** 10.1002/ece3.9920

**Published:** 2023-03-21

**Authors:** Xiaoyue Wang, Yan Chen, Yin Yi

**Affiliations:** ^1^ Key Laboratory of State Forestry Administration on Biodiversity Conservation in Karst Mountainous Areas of Southwestern China Guizhou Normal University Guiyang China; ^2^ School of Life Sciences Guizhou Normal University Guiyang China

**Keywords:** compatible pollination, distyly, floral scent composition, *Luculia pinceana*, nectar properties, pollinator

## Abstract

It is hypothesized that in heterostylous plant species, standardization of signals of floral attraction between different morphs is advantageous, encouraging flower visitors to switch between morphs. It remains unclear whether signals of floral attraction (floral odor and properties of nectar) are similar between morphs in distylous species pollinated by hawkmoths, and how these relate to hawkmoth behavior. We observed the behavior of visitors to distylous *Luculia pinceana* (Rubiaceae), collected and analyzed floral odor, and examined properties of nectar (volume, sugar concentration, and composition) of long‐styled and short‐styled morphs during the day and night. Pollinator responses to the floral scent were tested with a Y‐tube olfactometer. We conducted diurnal and nocturnal pollination treatments and six other pollination treatments to test the importance of nocturnal pollinators and to examine the self‐incompatibility system. A species of hawkmoth, *Cechenena lineosa*, was the effective pollinator. The floral odor was rich in methyl benzoate, and sucrose was dominant in the nectar. There were no significant differences between the two morphs in the methyl benzoate content or the properties of nectar. Flowers released more methyl benzoate and secreted larger volumes of nectar with lower sugar concentration at night than during the day. The hawkmoth had a significant preference for methyl benzoate. *Luculia pinceana* was partially self‐incompatible and relied on nocturnal pollinators for reproductive success. This study verifies that floral attraction signals are consistent between different morphs in this distylous species, promoting compatible pollination, and the features and the diel pattern of these signals between day and night are adapted to hawkmoth behavior.

## Graphical abstract

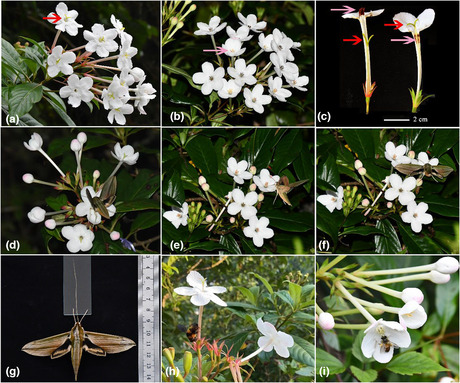

## 1 INTRODUCTION

Plant–pollinator interactions play an important role in shaping floral diversity in angiosperms (Fenster et al., 2004; Harder & Johnson, 2009). Heterostyly is a polymorphic system in which individual plants of a species have flowers of either of two (distyly) or three (tristyly) floral morphs. Distyly is usually accompanied by self‐incompatibility, which prevents self‐ and intramorph fertilization, and promotes intermorph pollination (transmission and receipt of pollen between anthers and stigmas at the same level, also called compatible pollination; Baker, 1964; Barrett, 2002; Darwin, 1877; Lloyd & Webb, 1992). Heterostylous plants commonly have narrow tubular corollas and achieve compatible pollination via long‐tongued pollinators, like bees, butterflies, and flies (Lloyd & Webb, 1992). Standardization of floral signals between different heterostylous morphs is advantageous, encouraging flower visitors to switch from one morph to another morph (reviewed in Lunau et al., 2017). If these signals differ between floral morphs (long‐styled or short‐styled morphs), driving floral visitors to one morph preferentially, more visitations will result in incompatible pollen transfer due to the self‐incompatibility of heterostylous species.

Floral scent is often a major floral signal attracting visiting animals (Byers, 2021; Raguso, 2008). The odor cues are especially obvious in nocturnal bat‐, moth‐, and hawkmoth‐pollinated flowers, and are also common in a broad range of bee‐pollinated flowers (Knudsen & Lars, 1993; Terry et al., 2021; Willmer, 2011). A few studies have compared floral scent signals between long‐styled (abbreviated as L‐morph) and short‐styled (abbreviated as S‐morph) morphs of distylous plants (Gaskett et al., 2005; Johnson, Golonka, et al., 2019). There was no significant difference in relative amounts of volatile compounds between the two morphs of *Primula elatior* or *P. farinosa* (Gaskett et al., 2005).

Plants reward pollinators with nectar (Andersson & Dobson, 2003; Baker, 1975; Pellmyr & Thien, 1986), as well as pollen, floral oil, etc. (Willmer, 2011). As another important floral attraction signal, nectar is derived from the phloem and is crucial for most pollinators, its sugars being simple to metabolize and thus to use as a readily available fuel for an animal's activities (de La Barrera & Nobel, 2004; Nicolson et al., 2007). Nectar properties, including nectar volume, sugar concentration, and sugar composition (Leiss & Klinkhamer, 2005; Mitchell, 2004), are subjected to pollinator‐mediated selection (Baker, 1975; Galetto et al., 2018; Potascheff et al., 2020; Vandelook et al., 2019). There was no significant difference in total sugar concentration between L‐ and S‐morphs of *Fagopyrum esculentum*, while the S‐morph secreted higher volumes of nectar than the L‐morph (Cawoy et al., 2008).

In tropical and temperate parts of the globe where dusk temperatures are high, hawkmoths are crucial for the compatible pollination of distylous plants (McMullen, 2012; Wang et al., 2022). Even in low light conditions, hawkmoths can accurately locate a flower and extend the proboscis into the base of the flower tube to extract nectar (Jürgens, 2004; Wiesenborn & Baker, 1990). In hawkmoth‐pollinated flowers, floral odors are generally dominated by terpenes, aromatic esters, and/or nitrogen compounds (Kaiser & Tollsten, 1995; Raguso et al., 2003) and hawkmoths have a preference for sucrose‐rich nectar (Nicolson et al., 2007). For example, the scent emission and mean number of scent compounds of African woodland orchid were highest at 19:30 h and the scent compounds differed between day and night. The nectar volume increased throughout the day and peaked just before the onset of pollinator activity, and the nectar was rich in sucrose (Balducci et al., 2020). *Qualea grandiflora* secreted nectar with a high proportion of sucrose and only at night, consistent with hawkmoths' foraging period and preference (Potascheff et al., 2020). These diel patterns and features of floral scent emission and nectar production of plants are adapted to hawkmoth behavior. However, it remains unclear whether the floral attraction signals (floral odor and nectar properties) of L‐ and S‐morphs in distylous hawkmoth‐pollinated plants are similar and how these traits are related to hawkmoth pollination.

The distylous *Luculia pinceana* (Rubiaceae) has a long narrow corolla tube with obvious nectar, white flowers, and strong fragrance. It is an ideal species for testing whether the floral odor and nectar properties are similar between two morphs and how these signals are adapted to hawkmoth behavior. This study aims to (1) explore the effective pollinator of the distylous *L. pinceana*; (2) identify the main floral odor compounds in the two morphs during the day and at night and test whether pollinators have a preference for the main odor compounds; (3) compare the nectar volume, sugar concentration, and sugar composition of the two morphs and compare the nectar volume and sugar concentration of the two morphs between day and night; (4) investigate the relative contribution to pollination of diurnal and nocturnal pollinators and examine the breeding system and test whether *L. pinceana* is self‐incompatible.

## 2 MATERIALS AND METHODS

### 2.1 Study materials and study sites

**FIGURE 1:**
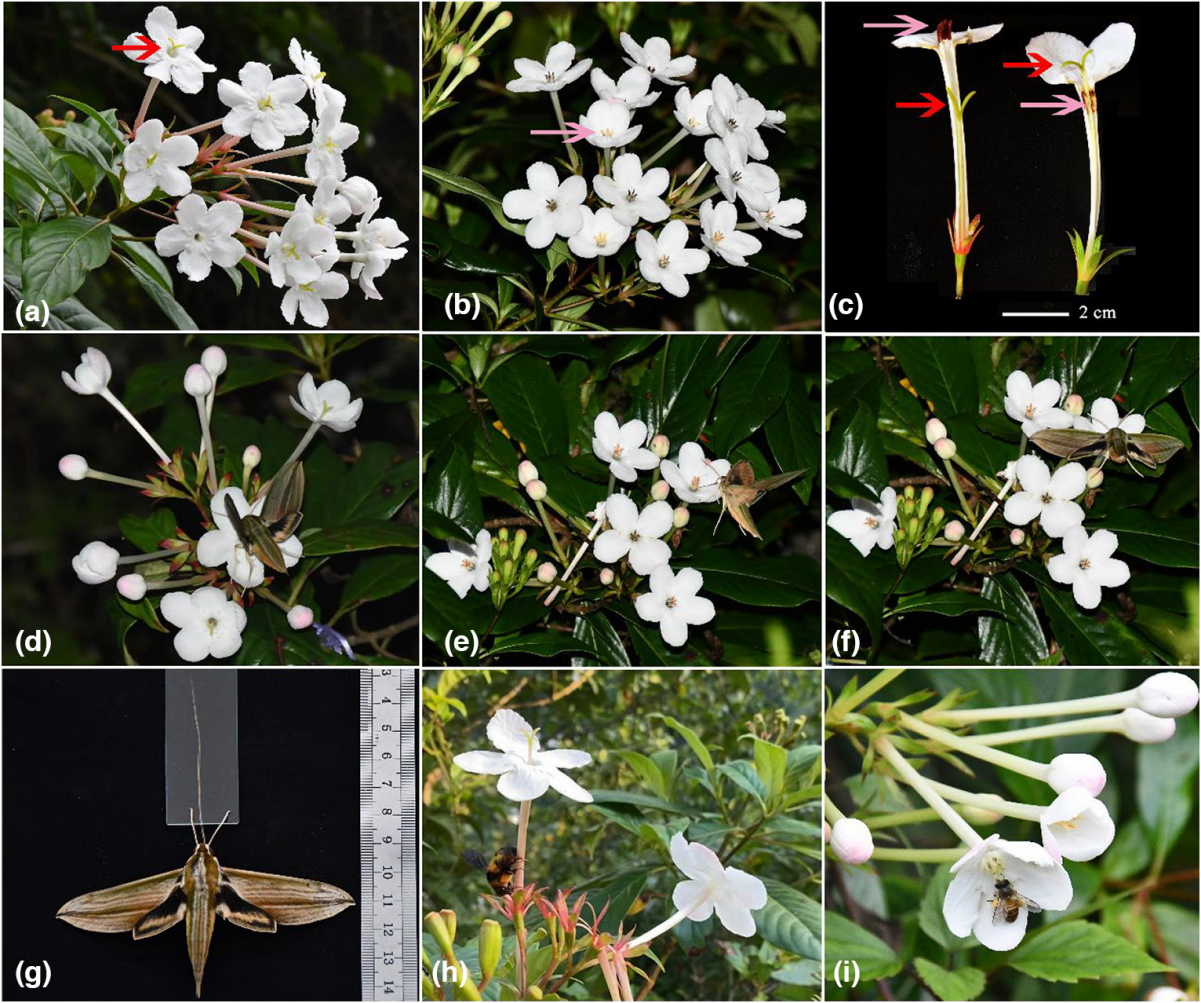
(a) The cymose inflorescence of the long‐styled morph (the long style marked with a red arrow) of *Luculia pinceana*; (b) the cymose inflorescence of the short‐styled morph (the long anther marked with a pink arrow) of *L. pinceana*; (c) flowers of the short‐styled morph (left) and long‐styled morph (right) showing the reciprocal positioning of stigmas (red arrows) and anthers (pink arrows); (d,e,f) the long–tongued *Cechenena lineosa* foraged for nectar; the tongue could touch the stigma and anther and pollinate the distylous *L. pinceana*; (g) the hawkmoth *C. lineosa*; the tongue length was about 6 cm which is mechanical fit with the *L. pinceana* floral tube; (h) a bumblebee robbing nectar from the bottom of the corolla tube; (i) a honeybee gathering pollen grains on a short‐styled inflorescence.

*Luculia pinceana* (Rubiaceae) is a perennial evergreen shrub or tree, 2–10 m tall, mainly distributed in forests or thickets on mountain slopes and streamside in valleys at an elevation of 600–3000 m in Yunnan and Guangxi provinces of China and in Thailand and Vietnam. The cymose inflorescence (Figure 1) is large and graceful, with a strong floral scent, and is generally terminal at the top of a stem or branch. The corolla is usually pink, sometimes white, and the corolla tube is cylindrical and glabrous. The stamens are inserted at the throat of the corolla tube at a level that depends on the morph, and the anthers are linear oblong. The flowering period generally lasts from June to December. The seeds are numerous, nearly elliptic, with wings at both ends. *Luculia pinceana* is a typical distylous plant with an L‐morph (with anthers low in the corolla and high stigmas) and an S‐morph (with anthers high and stigmas low; Figure 1a‐c).

The experimental site is Laoshan Provincial Nature Reserve (104°49′62″ E, 23°94′8″ N, approximately 1600 m above sea level) in Malipo County, Wenshan City, Yunnan Province, southwestern China. We obtained the *L. pinceana* plants and insects with permission from the Laoshan Provincial Nature Reserve, and the formal identification of the *L. pinceana* plants and visiting insects was undertaken by Liu Changqiu, associate researcher, Guangxi Institute of Botany, Chinese Academy of Science.

### 2.2 Pollinator observations

To determine the effective pollinator of *L. pinceana*, we observed all visits of different species for eight sunny days (June 22 and 26–30; July 1, 3) in 2019 and 12 sunny days (July 14, 16–18, 23, 24, 26, 27; August 1, 4, 5, 9) in 2020 at five plots (Table S1). Each session lasted for 30 min between 7:00 and 22:00. A total of 44 and 52 sessions were completed in 2019 and 2020, respectively. In each plot, we randomly chose about 50 blooming flowers from two or three individuals per morph. When one visitor came, the visit number per foraging bout, visitor species, and foraging behavior (for the nectar or pollen) were recorded, and the total open flowers in each plot were counted. The visit frequency of one visitor was equal to the mean number of visits per flower per hour. If the visiting insect extended its long tongue into the corolla tube and touched the anthers (removing pollen grains) and stigmas (depositing pollen grains), it was considered an effective pollinator. The species and behavior of visitors were recorded using cameras (Nikon D7500). The pollinators were considered effective not only based on their foraging behavior (extending its long tongue into the corolla tube and touching the anthers and stigmas) but also the mechanical fit between the hawkmoth tongue length and the floral tube length of *L. pinceana* (the data have been published in Chen et al., 2021). Sunrise was about 06:00 h and sunset was about 18:00 h during this period.

### 2.3 Floral odor collection and component analysis

To detect the floral odor composition of *L. pinceana* in the field, floral volatiles were collected using dynamic head‐space methods (Dotterl et al., 2005). Each sample contained 10 newly opened flowers and some leaves enclosed within a disposable polyethylene oven bag (Toppits) directly from the plant. Scent samples were collected from 10 plants at about 10: 00 h during the 2019 season. To further compare the main floral odor component (methyl benzoate) of two morphs in *L. pinceana* between day and night, 49 floral scent samples were collected (12 samples of the L‐morph and 15 samples of the S‐morph collected at 10:00 h; 11 samples of the L‐morph and 11 samples of the S‐morph collected at 20:00 h). The scent samples collected during the day and at night were from the same plant individuals. The emitted floral scent volatiles were trapped in an adsorbent glass tube using a membrane pump (ASF Thomas, Inc.). The flow rate was adjusted to approximately 200 mL/min using a power supply and a flow meter and each collection lasted 30 min. A transparent quartz glass test tube (length: 20 mm, inner diameter: 2 mm, Lianyungang Dong Hong Quartz Glass Company) was filled with Tenax‐TA powder (mesh 60–80) and used as the adsorbent tube. The adsorbent powder was fixed in the tube using glass wool (HJ637–2018). To control for scent from leaves and the ambient air, three samples were simultaneously taken from oven bags with branches including just leaves from three individuals (Feng et al., 2023). After collection, both ends of the adsorbent tube were sealed with plastic film, and the samples were placed into brown injection bottles and stored at −18°C for about 3 months.

In the laboratory, each sample was washed with a mixture of *n*–hexane (100 μL) and ether (100 μL), extracted ultrasonically for 30 min, and centrifuged at 8609 *g* for 3 min. The volume of each sample solution (mL) was recorded. The floral scent of *L. pinceana* was analyzed quantitatively on a Varian 450 gas chromatography (GC)‐Varian 320 TQ mass spectrometer (MS) on a Varian Saturn 2000 System. The GC was performed with Agilent 7694 E headspace samplers. The conditions of GC were as follows: Agilent 19,091 J ‐413 column (30.0 m long; inner diameter, 320 μm; film thickness, 0.25 μm; nonpolar); the starting temperature of 60°C was maintained for 3 min, increased from 20°C/min to 290°C, and kept for 1 min. The injection volume was 2 μL. Nondiversion mode was adopted. The injection inlet temperature was 280°C. The carrier gas was helium. The conditions of MS were as follows: the ion source temperature was 300°C, electron energy was 10 V at full scanning mode, the transmission line temperature was 200°C, and the mass spectrum scanning range was 40–650 amu. Floral scent components were identified by comparing their mass fragmentation with the data from the NIST 2008 mass spectral library (NIST/EPA/NIH Mass Spectral Library, data version NIST 2008, mainly including REPLIB, MAINLIB) and Wiley 6 electronic library (the cutoff match was 75%), referring to scientific literature about floral odor compounds in the Rubiaceae (Li et al., 2016, 2017; Zheng et al., 2020), and the description of the chemical compound properties on the chemical book website (http://www.chemicalbook. com). Most compounds are tentatively identified using these libraries and literature. Some matching chemical materials found by looking up the literature and chemical book website are not plant natural products and some have no certain CAS number. All these chemical materials were excluded from the analysis. To determine the relative amounts of floral odor compounds in the samples from 2019, peak areas were normalized as a percentage ((%) = peak areas of one volatile/peak areas of total volatiles * 100).

For further quantification of sample compounds known to be methyl benzoate, methyl benzoate standard solution of different concentrations (0.1, 0.5, 1.0, 10.0 mg/mL) was injected and analyzed. Methyl benzoate standard with a purity of 99% was purchased from Tianjin Kemiou Chemical Reagent Co., Ltd for calibration. Regressions based on the response peak area to the contents of each methyl benzoate standard solution were calculated. The coefficient of determination was 0.9995 or greater. The methyl benzoate content (mg/mL) in floral scent samples of 2020 was quantified by comparison with the standards using the regression equations.

### 2.4 Behavioral preference of pollinating hawkmoths

**FIGURE 2:**
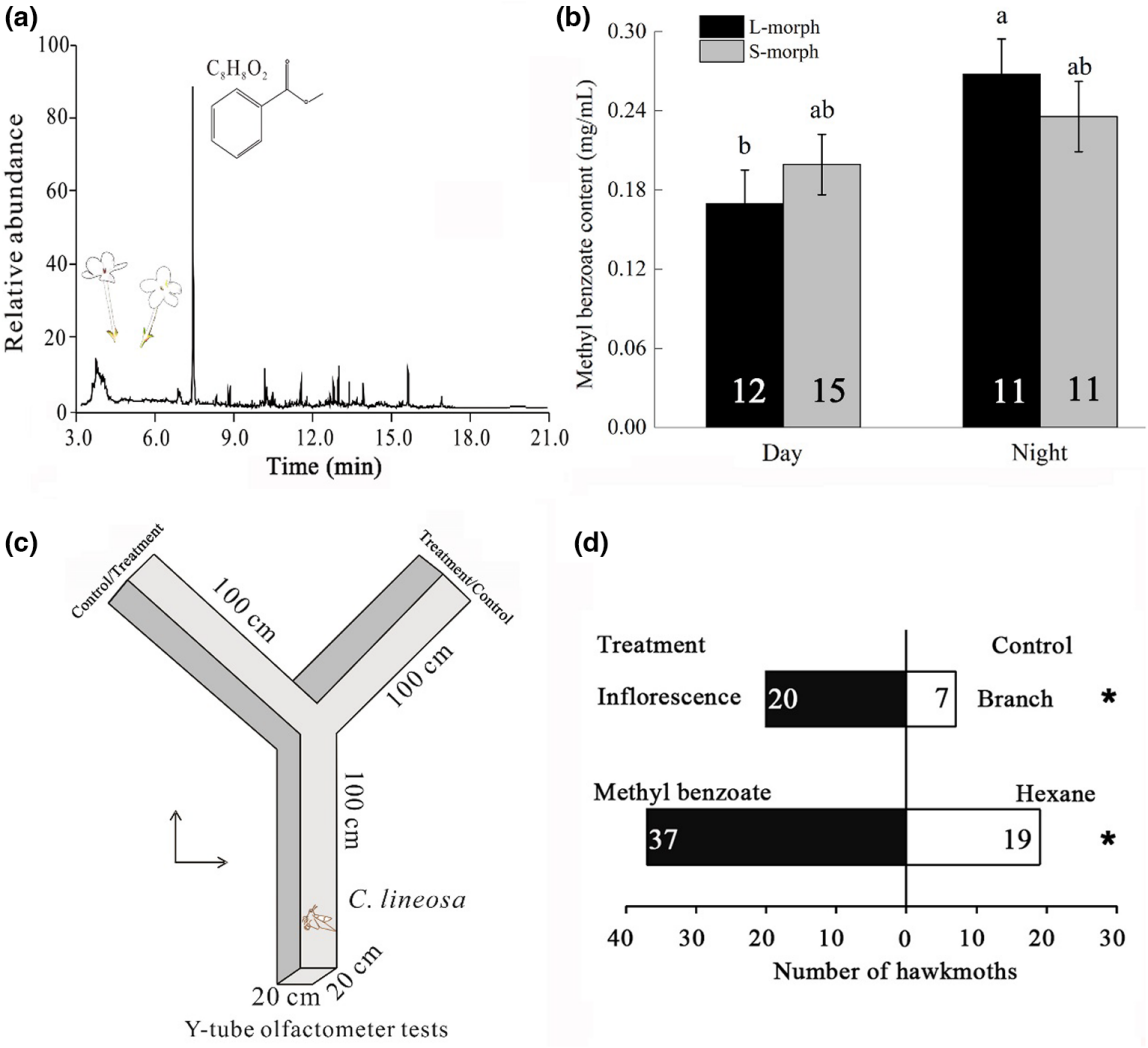
(a) Gas chromatography–mass spectrometry analysis of ion chromatogram of *Luculia pinceana* floral odor (main component: methyl benzoate, retention time: 7.428 min). (b) Comparison of methyl benzoate content between day and night in the two morphs of *L. pinceana*. The numbers in the bat charts are sample size and bars sharing the different letters are significantly different between treatments at the *p* < .05 level. (c) Schematic diagram of Y‐tube olfactometer. (d) The preference of the hawkmoth *Cechenena lineosa* for treatment and control ends in Y‐tube olfactometer tests. The numbers in columns are sample size, and * indicates a significant difference at the .05 level.

To examine whether the effective adult pollinators of *L. pinceana* have a preference for the main floral scent component, 60 hawkmoths were captured with high‐pressure mercury vapor lamps (450‐watt, Shanghai Yaming Factory) at night in Da Xi Factory, Yunling Village, Djinchang Town, Ma Lipo County (104°84′60″ E, 23°17′9″ N) in August 2020. After capture, the hawkmoths were tested immediately on the same night. The preliminary experiment showed that hawkmoths that were kept in a cage did not make a preference choice, perhaps because they were frightened in the breeding cage. When different odor sources were connected to each end of the Y‐tube olfactometer, the insects crawled toward the favorite odor. Considering all the features of the equipment and the purpose of this research, we designed a bespoke Y‐tube glass olfactometer with one vacuum pump, two drying towers, two flowmeters, and two gas washers (produced by Nanjing Shelly company). The Y‐tube had three channels each 100 cm long (Jarriault et al., 2009), with an inner diameter of 25 cm, schematically shown in Figure 2c. The wingspan of the hawkmoth was 73.13 ± 11.74 (mm), the body length was 49.12 ± 4.65 (mm; Chen et al., 2021). The channel was big enough for the hawkmoth to crawl and fly.

The methyl benzoate standard was diluted with *n*‐hexane to 0.2 mg/mL to correspond with the detected average content of methyl benzoate (the main chemical substance) in *L. pinceana* floral odor. The methyl benzoate and *n*‐hexane blend were randomly placed at one end of the Y‐tube olfactometer, and *n*‐hexane was placed at the other end as a control. Odors were passed from both arms to the stem in equal flow rates of cleaned and humidified air flow created by an air pump system (super silent positive pressure vacuum pump SCI‐HS25ZF: voltage 220 V, power 1.5 W, average flow 3 L/min, air pressure 0.016 MPA, pressure adjustable) via an activated charcoal filter and distilled water. To improve experimental operation without interfering with the hawkmoth's behavior, a small flashlight (KM‐8911 lamp, Kangming Company) covered with thick red plastic film (radiating faint red light) was placed more than 3 m away from the olfactometer and not directly exposed to it. The olfactometer was surrounded with a dark environment. Liu and Huang (2013) showed that a small flashlight covered with thick red plastic film could be used for nocturnal moth observations and would not disturb the moth's foraging behavior. Each hawkmoth was tested only once, and its behavior was assigned to one of the three choices: (1) flying completely to the floral scent source and staying at the end for more than 10 s; (2) flying to the control end; (3) if the hawkmoth flew irregularly for 10 min, it was deemed that no choice had been made (Wan et al., 2015). We repeated this experiment 60 times (i.e., 60 hawkmoths) and compared the number of different choices using a G‐test of goodness of fit to see whether the hawkmoths preferred one end to the other.

An inflorescence (with 10 flowers) and a branch (with 10 leaves) from the same plant of *L. pinceana* were picked and the inflorescence was randomly placed at one end of the Y‐tube olfactometer, and the branch was placed at the other end as control. In total, we got 30 groups of inflorescences and branches from different individuals. A total of 30 hawkmoths were captured and we tested the preference choice response to the inflorescence or branch with the methods described above. Each test used a new inflorescence and branch. During the experiment, the methyl benzoate or inflorescence (treatment) and the hexane or branch (control) were randomly placed on either side of the Y‐tube olfactometer. Each hawkmoth was tested only once. After testing and before testing the next hawkmoth, the olfactometer was cleaned firstly with 75% ethanol and then with distilled water, and dried with a clean dry cloth, to prevent the accumulation of odor. After each measurement, the tested hawkmoth was temporarily placed in a cage and then released back to the field.

### 2.5 Nectar volume, sugar concentration, and sugar composition of *Luculia pinceana*

To compare the nectar volume and sugar concentration from the same individual *L. pinceana* flower between day and night during anthesis, we bagged and labeled 50 buds with nylon mesh bags per morph from 50 L‐morph individuals and 50 S‐morph individuals. During blooming, the nectar in the bagged flower (on the first day of anthesis) was removed using glass microcapillary tubes (0.3 mm in diameter), which were inserted into the base of the corolla. The nectar volume secreted during the day (from 06:00 to 18:00 h) was measured at 18:00 h. The same flower was bagged again after the measurement. The nectar volume secreted from the same flower during the night (from 18:00 to 06:00 h the next day) was measured at 06:00 h the next day. Then, the length (L) of the microcapillary tube occupied by nectar was measured using a caliper micrometer (accurate to 0.01 mm). The nectar volume was equivalent to π * 0.15^2^ * L. The sugar concentration of each nectar sample was measured using a hand‐held refractometer (Eclipse 0%–50%; Bellingham and Stanley Ltd.) and the units of the nectar sugar concentration were g sucrose per 100 g solution, known as % Brix (Corbet, 2003). Three buds in L‐morph and four buds in S‐morph were not fully developed and we only get 47 samples for L‐morph and 46 samples for S‐morph.

To examine the composition of the nectar, we used 30 blooming flowers per morph from 30 individuals. The nectar of each flower was extracted with a capillary tube, and the nectar volume was measured. Then, the nectar was blown onto a filter paper and air‐dried at room temperature. The filter papers with nectar were placed in a 2‐mL centrifuge tube and stored in a −18°C refrigerator. Nectar samples were dissolved in 100 μL of deionized water for 24 h at room temperature before detection. The sugar contents of nectar (mainly glucose, fructose, sucrose, and maltose) were analyzed by high‐performance liquid chromatography (HPLC) with a refractive index detector and an Agilent Zorbax carbohydrate analysis column 843300–908 (Agilent Technologies). The mobile phase was an acetonitrile: water system (80:20 by volume) at a flow rate of 1 mL/min. The column temperature was 35°C, and the injection volume was 20 μL. The quantities of each sugar in nectar samples were calculated by comparison with standards (fructose, glucose, sucrose, and maltose) using the regression equation (based on response peak to standard sugar) and were expressed as relative percentages by mass (Sun et al., 2017).

### 2.6 Pollination treatments

To investigate the relative pollination contribution of diurnal or nocturnal pollinators of *L. pinceana* and test whether *L. pinceana* was self‐ and intramorph incompatible, we randomly chose 30 plants per morph and selected eight buds in each plant. Three of the eight buds were used for the pollination treatments: (1) Open pollinated treatment as control: flowers were always exposed; (2) nocturnal pollination: the flowers were bagged with nylon mesh bags from 06:00 to 18:00 h and exposed from 18:00 to 06:00 h the next day; (3) diurnal pollination: the buds were exposed from 06:00 to 18:00 h and bagged with nylon mesh bags from 18:00 to 06:00 h the next day. The pollination treatments continued until the flowers faded. The other five buds on each plant were labeled with cotton threads of different colors. Four of the five buds were emasculated and bagged with nylon mesh bags until they developed into the female phase and then received the following pollination treatments: (4) self‐pollination (using pollen from the flowers of the same individual); (5) test for apomixis (after emasculation, the flowers were always bagged); (6) intramorph pollination (pollen from flowers of the same morph); (7) intermorph pollination (pollen from flowers of the other morph). The last one bud was used as (8) autonomous self‐pollination treatments (the flowers were bagged until they withered; Liu & Huang, 2013; Wang et al., 2022).

Three months after pollination, the ripe fruits of eight pollination treatments were collected and the seeds were counted under a dissecting microscope. Seed set (%) equals the number of fertilized seeds/the number of fertilized and unfertilized seeds * 100%. The self‐incompatible (SI) index of *L. pinceana* was calculated (Ferrer et al., 2009) to estimate the strength of SI and categorize the degree of self‐incompatibility. SI index = ss_*i*_/ss_*o*_. ss_*i*_ is the mean seed set after self‐pollination in a plant and ss_*o*_ is the mean seed set after cross‐pollination in the same plant. For statistical analyses, plants were grouped by their SI index into the following four SI categories modified from Ruiz‐Zapata and Arroyo (1978): (1) strongly self‐incompatible (SI index = 0), (2) self‐incompatible (0 < SI index < 0.149), (3) partially self‐incompatible (0.15 < SI index < 0.49), and (4) self‐compatible (SI index > 0.5).

### 2.7 Data analysis

We compare the nectar volume/nectar sugar concentration between day and night (time factor), between L‐ and S‐morph (morph factor) in generalized linear model (GLM) with normal distribution and identity link function in SPSS 20.0, with nectar volume (μL)/nectar sugar concentration (%) as dependent variable, day and night as factor 1, L‐ and S‐morph as factor 2, and the interaction between factor 1 and 2 was also analyzed. To detect the difference of nectar volume/nectar sugar concentration between day and night in L‐morph/S‐morph, we compare the nectar volume/nectar sugar concentration between day and night in L‐morph/S‐morph in GLM with normal distribution and identity link function (nectar volume (μL)/nectar sugar concentration (%) in L‐morph/S‐morph as dependent variable, day and night as factor).The sugar composition was compared between the two morphs in GLM with normal distribution and identity link function (with glucose, fructose, and sucrose components (μg/μL) as dependent variables, and L‐ and S‐morphs as factors). The methyl benzoate amount between L‐and S‐morphs and between day and night was compared in GLM with normal distribution and identity link function (with methyl benzoate amounts (mg/mL) as the dependent variable, and day and night as factor 1, L‐ and S‐morphs as factor 2, the interaction between factor 1 and 2 was also analyzed). A G‐test of goodness of fit was used to see whether hawkmoth pollinators prefer one end over the other during the Y‐tube olfactometer test. The seed sets of different pollination treatments in L‐ and S‐ morph were compared using binary logistic analysis in GLM (with full seed number as the dependent variable, total seed number as trials variable, and different treatments as factor 1, two morphs as factor 2, the interaction between factor 1 and 2 was also analyzed). All data were analyzed in SPSS 20.0 (IBM Inc.) software.

## 3 RESULTS

### 3.1 Pollinator species

Field observations in 2019 and 2020 showed that the nocturnal hawkmoth *Cechenena lineosa* and diurnal bumblebees (*Bombus* spp.) and honeybees (*Apis* sp.) visited *L. pinceana*. The hawkmoth flew to the corolla and extended its long tongue to probe for nectar at the base of the narrow corolla tube. The tongue would touch the anther and stigma. The L‐ and S‐ morph pollen grains could be deposited on different parts of the tongue, causing intermorph pollination (Figure 1d‐g). The hawkmoth frequently visited the *L. pinceana* flowers at dusk from about 17:00–20:30 h in the experimental field sites. Bumblebees and honeybees visited flowers more frequently by day. Bumblebees usually climbed to the lower lip of the corolla, moved to the base of the corolla tube, and used their powerful mouthparts to pierce the corolla tube and obtain the nectar (Figure 1h). Only certain bumblebee species can do this; others just used the robbing holes to get nectar. Honeybees commonly visited only S‐morph flowers (the anthers and pollen were located higher and easily accessed) and rarely visited L‐morph flowers. During the visits, the honeybees usually groomed and gathered the pollen into the corbicula using their legs (Figure 1i). The visit rate (visits/flower/hour) of hawkmoths was 0.04 ± 0.06 (mean ± SE). The visit rate of bumblebees was 0.47 ± 0.06 and that of honeybees was 0.29 ± 0.09 (the visit rate data in 2019 and 2020 were combined and analyzed).

### 3.2 Floral odor composition

A total of 29 floral odor volatile compounds were detected in *L. pinceana* inflorescences and could be divided into eight different types: acids (about 2.99%, mean of the relative amount), alcohols (about 40.61%), aldehydes (about 0.94%), alkanes (5.18%), esters (about 34.06%), hydrazines (about 3.03%), phenol (about 2.16%), and terpenes (about 1.55%). Some iron peaks do not match chemicals (unknown compounds) during our analysis and the summary of these relative amount for these was 7.55 ± 0.35 (%). Methyl benzoate, 2,3‐butanediol, palmitic acid, acetic acid, hydrazide, (e)‐isoeugenol occurred in relatively large amounts (>2%) and were consistently abundant across samples in 2019 and 2020. The scent was dominated by methyl benzoate (C_8_H_8_O_2_; an ester; the relative amount was about 32%; Figure 2a). Methyl benzoate was not detected in the control floral odor samples and the control samples mainly matched 2,3‐butanediol (the relative amount was about 77.54%) and several alkane chemicals (the relative amount was about 10.65%). The summary of the relative amount of these unknown compounds in control samples was 9.55 ± 0.29 (%; Table 1).

**TABLE 1:** Volatile compounds identified of *Luculia pinceana* flower using GC–MS and the relative amount (%) and number of samples (*n*) that each compound found in floral odor (10 in total) and control samples (3 in total). The compounds are arranged by compound class and retention time.

Compounds	Retention time	CAS	Relative amount (%, *n*) in floral odor samples	Relative amount (%, *n*) in control samples
Acids				
Palmitic acid	12.98	057–10‐3	2.19 ± 0.67 (5)	1.631 ± 0.51(3)
Stearic acid	13.92	057–11‐4	0.80 ± 0.23 (6)	
Alcohols				
2,3‐Butanediol	3.739	513–85‐9	40.34 ± 4.35 (10)	77.54 ± 0.86 (3)
2‐Hexanol	4.283	626–93‐7	0.27 ± 0.12 (3)	
Aldehydes				
Phenylacetaldehyde	6.945	122–78‐1	0.94 ± 0.33 (3)	
Alkanes				
Decane,2,3,6‐Trimethyl‐	8.691	62238–12‐4	0.25 ± 0.15 (3)	
Dodecane, 3‐methyl‐	8.786	17312–57‐1	0.72 ± 0.13 (5)	0.74 ± 0.17 (3)
Heptadecane	8.789	629–78‐7	0.32 ± 0.18 (8)	
Dodecane	9.688	112–40‐3	0.34 ± 0.02 (8)	
Tetradecane	9.745	629–59‐4	0.38 ± 0.21 (7)	
Tridecane	9.993	629–50‐5	0.17 ± 0.05 (4)	
Pentadecane	10.053	629–62‐9	0.33 ± 0.19 (3)	0.409 ± 0.06 (2)
Octadecane	10.088	593–45‐3	0.27 ± 0.12 (7)	0.292 ± 0.03 (3)
Hexadecane,2,6,10,14‐tetramethyl‐	10.139	638–36‐8	0.06 ± 0.02 (5)	0.963 ± 0.01 (2)
Eicosane	10.259	112–95‐8	0.62 ± 0.21 (4)	0.736 ± 0.03 (3)
Hexadecane	10.311	544–76‐3	0.64 ± 0.46 (5)	2.6 ± 0.6 (3)
Tetradecane,2,6,10‐trimethyl‐	10.545	14,905–56‐7	0.34 ± 0.07 (3)	
Triacontane	10.733	638–68‐6	0.22 ± 0.14 (3)	
Heptadecane	10.956	629–78‐7	0.21 ± 0.07 (8)	0.333 ± 0.07 (3)
Tricosane	10.99	638–67‐5	0.19 ± 0.05 (5)	1.868 ± 0.13 (3)
Nonadecane	11.508	629–92‐5	0.12 ± 0.02 (3)	
Esters				
Methyl benzoate	**7.429**	**93–58‐3**	**31.98 ± 0.30 (8)**	
Methyl salicylate	8.323	119–36‐8	0.35 ± 0.20 (3)	
Dioctyl phthalate	15.7	117–81‐7	0.12 ± 0.02 (3)	
Monostearin	16.91	123–94‐4	1.61 ± 0.87 (8)	1.085 ± 0.17 (3)
Hydrazines				
Acetic acid, hydrazide	6.294	1068‐57‐1	3.03 ± 0.58 (8)	
Phenols				
(e)‐isoeugenol	10.17	5932‐68‐3	2.16 ± 0.06 (3)	
Terpenes				
3,7,7‐trimethyl‐Bicyclo [4.1.0] hept‐3‐ene	6.858	13466–78‐9	1.33 ± 0.72 (3)	
16‐Hexadecanoyl hydrazide	8.529	2619‐88‐7	0.22 ± 0.12 (6)	
Iron peak with no chemical match	3.203			0.361 ± 0.01 (3)
	3.42			0.452 ± 0.01 (3)
	3.508		1.49 ± 0.06 (9)	1.146 ± 0.02 (3)
	4.205		0.53 ± 0.01 (10)	
	4.24		0.65 ± 0.02 (8)	0.17 ± 0.01 (3)
	4.334		0.62 ± 0.02 (10)	0.312 ± 0.02 (3)
	4.696		0.22 ± 0.01 (9)	0.147 ± 0.01 (3)
				
	4.798		0.56 ± 0.01 (9)	
	4.835		0.26 ± 0.01 (10)	
	5.019		0.30 ± 0.02 (8)	
	5.634		0.47 ± 0.02 (10)	
	6.903		1.1 ± 0.11 (10)	0.486 ± 0.01 (3)
	7.516			1.012 ± 0.01 (3)
	7.637		0.70 ± 0.02 (9)	1.002 ± 0.02 (3)
	7.845			0.594 ± 0.01 (3)
	8.216			1.406 ± 0.02 (3)
	9.984			0.88 ± 0.02 (3)
	10.485		0.65 ± 0.02 (8)	0.90 ± 0.02 (3)
	13.391			0.792 ± 0.01 (3)

*Note*: The significance of bold values is that the variation between different treatments has significant difference.

Generally, there was no significant difference in the methyl benzoate content between L‐morphs (0.22 ± 0.02 mg/mL, *n* = 23) and S‐morphs (0.21 ± 0.01 mg/mL, *n* = 26) flowers (the methyl benzoate content data of day and night of each morph combined together and analyzed), but significantly more methyl benzoate was released at night (0.25 ± 0.02 mg/mL, *n* = 22) than by day (0.19 ± 0.01 mg/mL, *n* = 27; the methyl benzoate content data of L‐ and S‐morphs during the day or at night were combined together and analyzed; Table 2). There was no interaction between floral morphs and emission times with respect to the methyl benzoate content (Table 2). The L‐morph inflorescences released significantly more methyl benzoate at night than during the day (1.59 times as much; the mean methyl benzoate content at night divided by that during the day, Wald *χ* ^2^ = 5.290, df = 1, *p* = .021), whereas in the S‐morph, there was no significant difference between night and day in the amounts of methyl benzoate released (Wald *χ* ^2^ = 1.519, df = 1, *p* = .218). During the day, there was no significant difference between the two morphs in the methyl benzoate content (Wald *χ* ^2^ = 0.437, df = 1, *p* = .508). The same was true at night (Wald *χ* ^2^ = 1.581, df = 1, *p* = .209; Figure 2b).

**TABLE 2:** Generalized linear model: effects of time (day vs. night), morphs (L‐ or S‐morph), and their interaction on methyl benzoate content, nectar volume, and sugar concentration in *Luculia pinceana*; effect of types (glucose, fructose, or sucrose), morphs, and their interaction on the nectar sugar concentration; effect of pollination treatments (control, nocturnal and diurnal pollination treatments, self‐pollination, apomixis, intramorph pollination, intermorph pollination, autogamy pollination treatments) and pollen recipient morph (L‐ or S‐morph) and their interaction on the seed sets of *L. pinceana.*

Source of variation	Wald *χ* ^2^	df	*p*
Methyl benzoate content			
Time (day or night)	6.732	1	**.009**
Morphs (L‐ or S‐morph)	0.005	1	.944
Interaction	1.469	1	.225
Nectar volume			
Time (day or night)	8.818	1	**.003**
Morphs (L‐ or S‐morph)	1.709	1	.191
Interaction	11.409	1	**.01**
Nectar sugar concentration			
Time (day or night)	6.001	1	**.014**
Morphs (L‐ or S‐morph)	0.005	1	.944
Interaction	7.64	1	.054
Nectar sugar composition			
Types (Glucose, fructose, or sucrose)	480.02	2	**<.001**
Morphs (L‐ or S‐morph)	0.739	1	.39
Interaction	1.166	2	.588
Seed sets			
Pollination treatments	411.51	1	**<.001**
Pollen recipient morph	17.167	7	**<.001**
Interaction	10.274	7	.174

*Note*: The significance of bold values is that the variation between different treatments has significant difference.

### 3.3 Preference of pollinators on floral scent

During the Y‐tube olfactometer test, the number of *C. lineosa* individuals flying to the methyl benzoate end (37) was significantly higher (*p* = .022, *G* = 5.243) than the number flying to the control end (19), and four *C. lineosa* did not make a choice. The number of *C. lineosa* flying to the inflorescence end (20) was significantly higher (*p* = .019, *G* = 5.524) than the number flying to the branch (as control) end (7), and three *C. lineosa* did not make a choice (Figure 2d). These results indicated that the effective pollinator *C. lineosa* had a significant preference for the methyl benzoate and the *L. pinceana* inflorescence.

### 3.4 Nectar volume, sugar concentration, and composition

**FIGURE 3:**
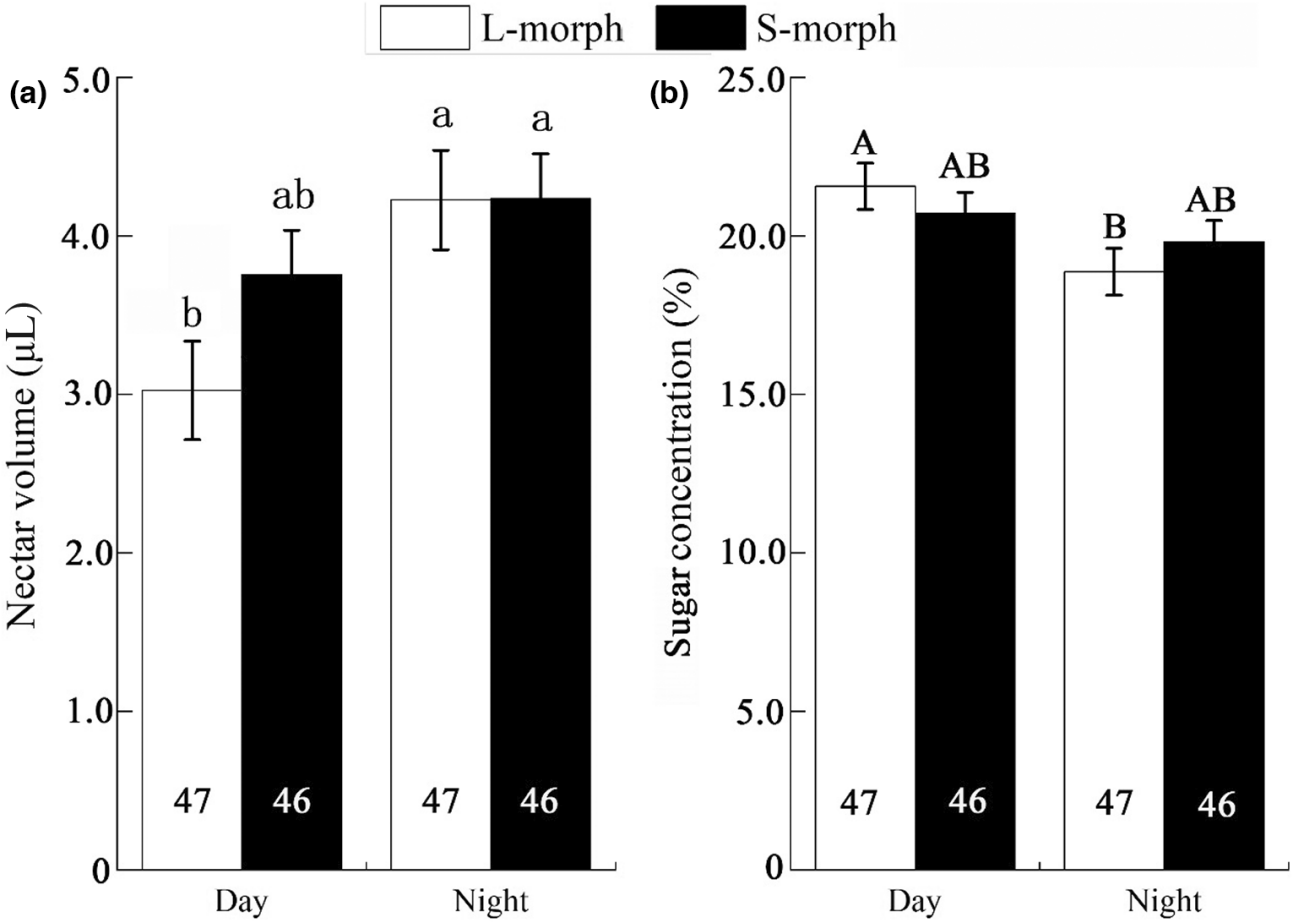
Comparison of the nectar volume and sugar concentration of two morphs in *Luculia pinceana* between day and night. The values in the bar charts represent sample size. The different letters indicate significant differences between the two morphs.

Generally, there was no significant difference in nectar volume between L‐ (3.63 ± 0.24 μL) and S‐ morphs (3.99 ± 0.17 μL) in *L. pinceana* flowers (Wald *χ* ^2^ = 1.709, df = 1, *p* = .191). The same was true for the nectar sugar concentration (L‐ morph: 20.23 ± 0.57%, S‐morph: 20.27 ± 0.44%, Wald *χ* ^2^ = 0.005, df = 1, *p* = .944). *Luculia pinceana* produced significantly larger volumes (1.23 times; the mean nectar volume at night divided by that during the day, Wald *χ* ^2^ = 8.818, df = 1, *p* = .003) but lower sugar concentrations (0.92 times; the mean sugar concentration at night divided by that during the day, Wald *χ* ^2^ = 6.001, df = 1, *p* = .014) at night than during the day (Figure 3; Table 2).

Moreover, the two morphs differed in the pattern of nectar volume and sugar concentration secreted during the day and at night. The L‐morph secreted a significantly larger volume at night than by day (1.40 times as much; the mean nectar volume at night divided by that during the day, Wald *χ* ^2^ = 3.828, df = 1, *p* = .01) but at a lower sugar concentration (0.88 times; the mean of sugar concentration at night divided by that during the day, Wald *χ* ^2^ = 5.878, df = 1, *p* = .015). For the S‐ morph, there was no significant difference between day and night in volume (Wald *χ* ^2^ = 1.962, df = 1, *p* = .161) or sugar concentration (Wald *χ* ^2^ = 1.045, df = 1, *p* = .307; Figure 3).

**FIGURE 4:**
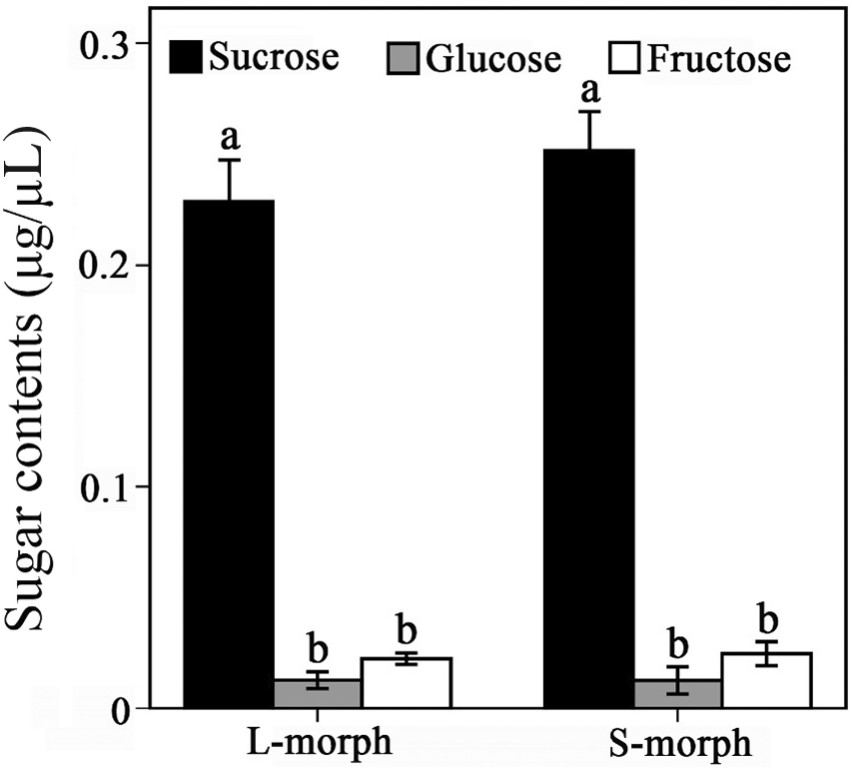
Comparisons of nectar sugar contents (sucrose, fructose, and glucose) in the two morphs of *Luculia pinceana*. The different letters indicate significant differences among the contents.

Glucose, fructose, and sucrose were detected in *L. pinceana* nectar by HPLC. In general, there was no significant difference between L‐ and S‐ morph in nectar sugar composition. The sucrose content (0.25 ± 0.01 μg/μL) was significantly higher than the glucose (0.03 ± 0.01 μg/μL) and fructose (0.01 ± 0.01 μg/μL) contents. There was no interaction between floral morphs and nectar sugar types with respect to the nectar sugar composition (Table 2). Sucrose content was significantly higher than glucose and fructose contents in L‐morph flowers (Wald *χ* ^2^ = 210.984, df = 2, *p* < .001) and in S‐morph flowers (Wald *χ* ^2^ = 278.277, df = 2, *p* < .001; Figure 4).

### 3.5 Pollination treatments

Seed sets of the L‐ (43.32 ± 3.03%) as pollen recipient were 1.41 times higher than seed set of S‐morph as pollen recipient (30.68 ± 2.72%). The seed sets of different pollination treatments differed significantly and there was no interaction between pollination treatments and pollen recipient morph with respect to seed set (Table 2).

**FIGURE 5:**
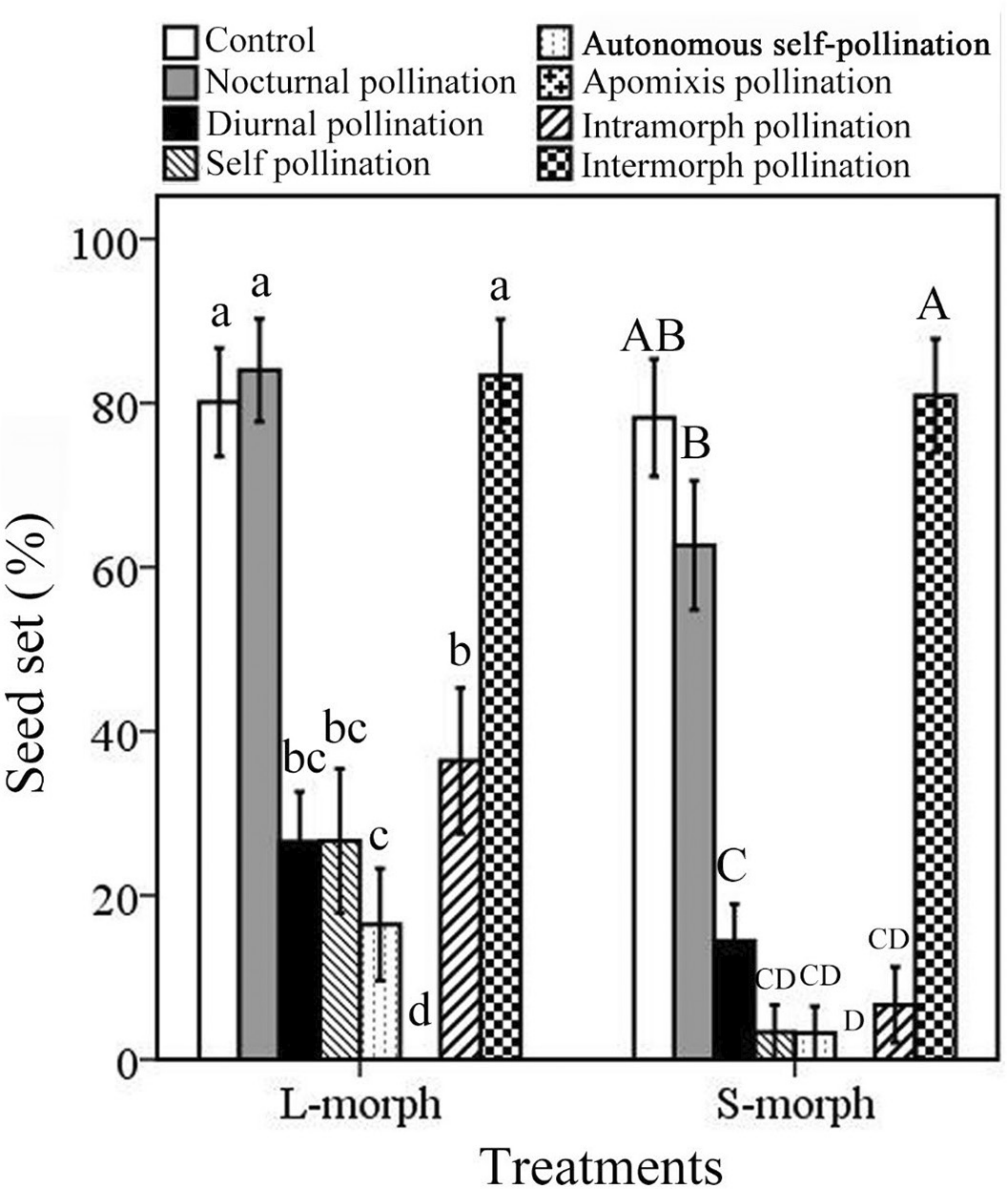
Comparisons of the seed sets (%) of control (referring to open pollination treatment, nocturnal, diurnal, self‐pollination, autonomous self‐pollination, apomixis, intramorph and intermorph pollination treatments of two morphs in *Luculia pinceana*. The different letters indicate significant differences among the different pollination treatments. The capital (A/B) letters and lowercase (a/b) letters, respectively, referred to the comparison of different pollination treatments in the S‐morph and the L‐morph as pollen recipient.

Generally, there was no significant difference in seed set between nocturnal pollination treatment (72.58 ± 5.24%) and open‐pollinated control treatment (79.13 ± 4.85%; Wald *χ* ^2^ = 0.832, df = 1, *p* = .362), and all were significantly higher than diurnal pollination treatments (19.91 ± 3.72%; all *p* < .05; seed set data of L‐morphs and S‐morphs as pollen recipients were combined and analyzed; Figure 5). For L‐morphs as pollen recipients, there was no significant difference in the seed set between nocturnal pollination and the open‐pollinated treatment (Wald *χ* ^2^ = 0.182, df = 1, *p* = .669), and both were significantly higher (Wald *χ* ^2^ = 55.511, df = 2, *p <* .001) than those of diurnal pollination treatment. The same was true for the S‐morph (Wald *χ* ^2^ = 63.377, df = 2, *p* < .001). The seed sets of nocturnal pollination in S‐morph (62.65 ± 7.89%) were lower than that of open pollination treatment (78.22 ± 7.15%) and they had no significant difference (Wald *χ* ^2^ = 1.101, df = 1, *p* = .294; Figure 5).

The seed set of intermorph pollination (82.09 ± 4.83%) for *L. pinceana* was significantly higher (Wald *χ* ^2^ = 240.575, df = 4, *p* < .001) than that of intramorph pollination (21.53 ± 5.33%), self‐pollination (14.15 ± 4.68%), autonomous self‐pollination (9.84 ± 3.85%), and apomixis (0.00 ± 0.00%). The difference between the seed sets of intermorph pollination and open‐pollinated treatment (79.13 ± 4.85%) was not significant (Wald *χ* ^2^ = 0.185, df = 1, *p* = .667; the seed set data of L‐morph and S‐morph as pollen recipient were combined and analyzed). For L‐morphs (as pollen recipients), there was no significant difference in seed set between the intermorph and open‐pollinated treatments (Wald *χ* ^2^ = 0.166, df = 1, *p* = .733), and both were significantly higher (Wald *χ* ^2^ = 123.546, df = 5, *p* < .001) than that of intramorph, self‐, autonomous self‐, and apomixis. The same was true for the S‐morph (Wald *χ* ^2^ = 0.072, df = 1, *p* = .788; Wald *χ* ^2^ = 319.790, df = 5, *p* < 0.001; Figure 5).

Moreover, there was no significant difference in seed set among open‐pollinated treatments (79.13 ± 4.85%), nocturnal pollination treatments (72.58 ± 5.24%), and intermorph pollination treatments (82.09 ± 4.83%; Wald *χ* ^2^ = 2.068, df = 2, *p* = .356; the seed set data of L‐morphs and S‐morphs as pollen recipients were combined and analyzed). There was no significant difference in seed set among open‐pollinated, nocturnal, and intermorph pollination in the L‐morph (Wald *χ* ^2^ = 0.169, df = 2, *p* = .919) or the S‐morph (Wald *χ* ^2^ = 3.667, df = 2, *p* = .160; Figure 5).

The ss_i_ of *L. pinceana* was 14.15. The mean seed set of intermorph cross‐pollination of *L. pinceana* was 82.09. It is well known that heterostylous plants are usually within‐morph cross‐incompatible. The ss_o_ was 82.09. The SI index of *L. pinceana* = 0.17 (14.15/82.09). The *L. pinceana* is partially self‐incompatible.

## 4 DISCUSSION

Our study showed that the distylous *L. pinceana* was pollinated by the long‐tongued hawkmoth *C. lineosa* and relied on nocturnal insects for reproductive success. The two morphs did not differ significantly in the main floral odor component (methyl benzoate) or properties of nectar (nectar volume, sugar concentration, composition). The floral attraction signal, including the larger content of methyl benzoate in the floral odor, the higher nectar volume and the relatively lower nectar sugar concentration at night than those during the day, and a sucrose‐dominated nectar sugar composition, were suggested to be ecologically adapted to hawkmoth. The hawkmoth *C. lineosa* had significant preference for the methyl benzoate odor and *L. pinceana* inflorescence. *Luculia pinceana* was partially self‐incompatible and the seed number of intermorph pollination was significantly higher than that of intramorph pollination.

Floral scents are important attractants for pollinators (Andersson, 2003; Johnson, Golonka, et al., 2019; Pellmyr, 1986). Hawkmoths can detect a variety of floral compounds, especially oxidized aromatic compounds, and they exhibit behavioral responses to many of these compounds (Balao et al., 2011; Johnson, Balducci, & Shuttleworth, 2019; Miyake & Yahara, 1998; Raguso et al., 1996). Linalool is the most common attractant for nocturnal hawkmoths in the floral scent of *Lonicera japonica*, and there is a significant correlation between the visitation of hawkmoths and specific floral odor (Miyake et al., 1998). The scent of *Dianthus inoxianus* is dominated by aliphatic 2‐ketones that contribute to attracting hawkmoth pollinators (Balao et al., 2011) and the nocturnal hawkmoth *Deilephila elpenor* responds to the odor of available extract of lavender positively (Balkenius et al., 2006). The hawkmoth *Manduca sexta* learns to feed from *Agave palmeri* flowers through olfactory conditioning but prefers *Datura wrightii* flowers based on an innate odor preference (Riffell et al., 2008). We found that the methyl benzoate content of *L. pinceana* floral odor was higher during the night than by day, and the hawkmoth (*C. lineosa*) was attracted to the methyl benzoate and made a behavioral preference response. Methyl benzoate is an important constituent in the floral odor of hawkmoth‐pollinated plants (Manning & Snijman, 2002; Raguso et al., 2003; Schlumpberger & Raguso, 2008) and has been shown to elicit strong antennal responses in two hawkmoth species *Manduca sexta* (Hoballah et al., 2005) and *Hyles lineata* (Raguso 1996).

Some previous research indicates that L‐morph flowers are significantly more strongly scented than S‐morph flowers which is consistent with the hypothesis that small flowers (L‐morph flowers) produce a stronger scent to defend against herbivory or attract pollinators (Leege & Wolfe, 2002). There was no significant difference in the floral odor between the two morphs in *Primula elatior* and *P. farinosa* (Gaskett et al., 2005). It is reasoned that heterostylous flowers are self‐incompatible and rely on pollinators visiting both morphs equally. The content of methyl benzoate, the main odor compound, did not differ significantly between two morphs of *L. pinceana* and this may contribute to attracting the hawkmoths to visit the two morphs equally, promoting compatible pollination.

Nectar properties and the different strategies of nectar presentation could influence the visiting behavior of floral visitors, thus affecting the reproductive success of plants (Hodges, 1995; Pacini et al., 2003). Nectar volume and sugar concentration may vary significantly within a day, depending on the physiological condition of the plant, the evaporation of water and collection by visiting insects (Angela et al., 2010; Corbet, 1978; Hagler & Buchmann, 1993). Nectar volume plays a major role in regulating the behavior of floral visitors (Nicolson et al., 2007). The hawkmoth pollinators visit significantly more *Mirabilis multiflora* flowers with larger amounts of nectar by manipulating the nectar volume (Hodges, 1995). A South American Andean cactus (*Echinopsis ancistrophora*) with high nectar volume is pollinated by hawkmoths (Schlumpberger et al., 2009). The hawkmoth pollinators reduce the probing duration on low‐nectar mutational *Petunia axillaris* individuals when they are exposed simultaneously to mutational and wild‐type *P. axillaris* (Anna et al. 2012). Both L‐ and S‐morph of *Tirpitzia sinensis* secrete larger nectar volume at night than by day which is potentially adaptive to hawkmoth pollination (Wang et al., 2022). *Luculia pinceana* secreted a larger volume of nectar with a lower sugar concentration during the night than by day which might be a direct dilution effect of nectar and the dilution might result from the higher relative humidity at night and are related to hawkmoth behaviors. The high nectar volume reflects the plant's resource investment in pollinator attraction.

Pollinators have different preferences for nectar sugar composition (Rio et al., 1992; Schondube & Rio, 2004). Nectar is mostly composed of fructose, glucose, and sucrose (Wykes, 1952). Moreover, nectar components vary significantly among plant species, probably to attract different pollinator species (Cruden et al., 1983). A recent study investigating the pollinator spectra of 57 Balsaminaceae species showed that the combination of nectar features reflects the pollination syndromes of flies, bird, bee, and butterfly pollination (Vandelook et al., 2019). Long‐tongued bees, butterflies, hawkmoths, and hummingbirds have a preference for sucrose‐rich nectar, while short‐tongued bees and flies prefer hexose nectar (Goodwin et al., 2011; Nicolson et al., 2007). According to Baker and Baker (1983), the ratio (equal to [amounts of sucrose/(amounts of fructose + amounts of glucose)]) of three common sugars in nectar could be divided into four types: sucrose dominant, sucrose rich, hexose rich, and hexose dominant. The nectar of the L‐morph (*r* = 5.75) and the S‐morph (*r* = 6.75) of *L. pinceana* is sucrose dominant. Plants with a large nectar volume, low sugar concentration, and high sucrose–hexose ratio are mainly adapted to hawkmoth pollination (Kristina et al., 2015).

Heterostylous plants are usually self‐incompatible and within‐morph cross‐incompatible (Armbruster et al., 2006; Barrett, 2019; Keller et al., 2014; Wolfe et al., 2009; Wu et al., 2018). For example, the distylous *Sebaea grandis* did not produce seeds under intramorph pollination treatment, while the seed set with intermorph pollination treatment was about 78% (Wolfe et al., 2009). Moreover, the incompatibility system of *Erythroxylum* species changed with deviations from a 1:1 ratio of L‐ and S‐morphs (Matias et al., 2020). The distylous species *L. pinceana* is partially self‐incompatible; the seed set of the intermorph pollination treatment in *L. pinceana* (about 77%) was significantly higher than that of the intramorph pollination treatment (about 17%) and the numbers of L‐ and S‐ morph individuals of *L. pinceana* in five field populations did not deviate from 1:1 (Chen et al., 2021).

## 5 CONCLUSION

The floral traits of the distylous *L. pinceana* (narrow floral tube, nectar, and floral scent traits) are typically adapted to hawkmoth pollination. *Luculia pinceana* secreted more nectar and emitted stronger floral scent at night which was remarkably attractive to *Cechenena lineosa*. *C. lineosa* is active during the night and the long tongue can touch the stigma and anthers of *L. pinceana* causing intermorph pollination. There was no significant difference in seed set between open pollinated and intermorph pollination treatments which indicates that *L. pinceana* receives enough legitimate pollen (intermorph pollen) for reproductive success. Moreover, there was no significant difference in seed set between nocturnal pollination and open pollinated treatments in *L. pinceana* and both were significantly higher than that with the diurnal pollination treatments. The hawkmoth contributes to compatible pollination. Although this work illustrates the relationship between the floral scent composition of *L. pinceana* and pollinator behavior, the effects of different compositions and concentrations of floral scent on pollinator behavior remain unknown, and further research is needed.

## AUTHOR CONTRIBUTIONS

**Xiao Yue Wang:** Conceptualization (lead); data curation (equal); formal analysis (equal); funding acquisition (lead); resources (lead); writing – original draft (equal); writing – review and editing (lead). **Yan Chen:** Data curation (equal); formal analysis (equal); methodology (equal); writing – original draft (equal); writing – review and editing (equal). **Yin Yi:** Formal analysis (equal); funding acquisition (equal); methodology (equal); writing – review and editing (equal).

## FUNDING INFORAMTION

This research was funded by grants from the National Natural Science Foundation of China (31901208); the Joint Fund of the National Natural Science Foundation of China and the Karst Science Research Center of Guizhou Province (U1812401); the Science and Technology Foundation of Guizhou Province (2019/1237); and new seeding plants project of Guizhou Normal University (grant No. 2020).

## CONFLICT OF INTEREST STATEMENT

The authors declare no competing interests.

## Supporting information


Table S1

## Data Availability

The data are available at the Dryad Digital Repository: 10.5061/dryad.kprr4xh7z.

## References

[ece39920-bib-0101] Andersson, S. (2003). Antennalresponses to floral scents in the butterflies *Inachis io, Aglaisurticae* (Nymphalidae), and *Gonepteryx rhamni* (Pieridae). Chemoecology, 13, 13–20.

[ece39920-bib-0102] Andersson, S. , & Dobson, H. E. D. (2003). Antennal responses to floral scents in the butterfly *Heliconius melpomene* . Journal of Chemical Ecology, 29, 2319–2330. 14682514 10.1023/a:1026278531806

[ece39920-bib-0001] Angela, K. , Luke, V. , McWhorter, T. J. , & Nicolson, S. W. (2010). Energy management on a nectar diet: Can sunbirds meet the challenges of low temperature and dilute food? Functional Ecology, 24, 1241–1251.

[ece39920-bib-0002] Armbruster, W. S. , Pérez‐Barrales, R. , Arroyo, J. , & Vargas, E. P. (2006). Three‐dimensional reciprocity of floral morphs in wild flax (*Linum suffruticosum*): A new twist on heterostyly. New Phytologist, 17, 581–590. 10.1111/j.1469-8137.2006.01749.x 16866960

[ece39920-bib-0003] Baker, H. G. (1964). Variation in style length in relation to outbreeding in *mirabilis* (Nyctaginaceae). Evolution, 18, 507–509.

[ece39920-bib-0004] Baker, H. G. (1975). Sugar concentrations in nectars from hummingbird flowers. Biotropica, 7, 37–41.

[ece39920-bib-0005] Baker, H. G. , & Baker, I. (1983). Floral nectar sugar constituents in relation to pollinator type. In R. J. Little & C. E. Jones (Eds.), Handbook of pollination biology (pp. 117–141). Scientific and Academic Editions.

[ece39920-bib-0006] Balao, F. , Herrera, J. , Talavera, S. , & Dötterl, S. (2011). Spatial and temporal patterns of floral scent emission in *dianthus inoxianus* and electroantennographic responses of its hawkmoth pollinator. Phytochemistry, 72, 601–609. 21376355 10.1016/j.phytochem.2011.02.001

[ece39920-bib-0007] Balducci, M. G. , Niet, T. V. , & Johnson, S. D. (2020). Diel scent and nectar rhythms of an African orchid in relation to bimodal activity patterns of hawkmoth pollinators. Annals of Botany, 126, 1155–1164. 32674148 10.1093/aob/mcaa132 PMC7684705

[ece39920-bib-0008] Balkenius, A. , Rosén, W. , & Kelber, A. (2006). The relative importance of olfaction and vision in a diurnal and a nocturnal hawkmoth. Journal of Comparative Physiology B, 192, 431–437. 10.1007/s00359-005-0081-6 16380841

[ece39920-bib-0010] Barrett, S. C. H. (2002). The evolution of plant sexual diversity. Nature Reviews Genetics, 3, 274–284. 10.1038/nrg776 11967552

[ece39920-bib-0011] Barrett, S. C. H. (2019). 'a most complex marriage arrangement': Recent advances on heterostyly and unresolved questions. New Phytologist, 224, 1051–1067. 31631362 10.1111/nph.16026

[ece39920-bib-0012] Byers, K. J. R. P. (2021). "As if they discovered it by the scent": Improving our understanding of the chemical ecology, evolution, and genetics of floral scent and its role in pollination. American Journal of Botany, 108, 729–731. 34008177 10.1002/ajb2.1661

[ece39920-bib-0013] Cawoy, V. , Kinet, J. M. , & Jacquemart, A. L. (2008). Morphology of nectaries and biology of nectar production in the distylous species *Fagopyrum esculentum* . Annals of Botany, 102, 675–684. 18765442 10.1093/aob/mcn150 PMC2712373

[ece39920-bib-0015] Chen, Y. , Wang, S. Y. , You, X. S. , Hu, D. M. , Yao, R. X. , Tang, X. X. , & Wang, X. Y. (2021). A study of pollination accuracy in distylous Rubiaceae species (*Luculia pinceana*). Acta Ecologica Sinica, 41, 6654–6664 (In Chinese with English abstract).

[ece39920-bib-0016] Corbet, S. A. (1978). Bee visits and the nectar of *Echium vulgare* L. and *Sinapis alba* L. Ecological Entomology, 3, 25–37.

[ece39920-bib-0017] Corbet, S. A. (2003). Nectar sugar content: Estimating standing crop and secretion rate in the field. Apidologie, 34, 1–10.

[ece39920-bib-0018] Cruden, R. W. , Hermann, S. M. , & Peterson, S. (1983). Patterns of nectar production and plant‐pollinator coevolution. In B. Bentley & T. Elias (Eds.), The biology of nectaries (pp. 80–125). Columbia University Press.

[ece39920-bib-0019] Darwin, C. (1877). The different forms of flowers on plants of the same species. John Murray.

[ece39920-bib-0020] de La Barrera, E. , & Nobel, P. S. (2004). Nectar: Properties, floral aspects and speculations on origin. Trends in Plant Science, 9, 65–69. 15102371 10.1016/j.tplants.2003.12.003

[ece39920-bib-0021] Dotterl, S. , Wolfe, L. M. , & Jürgens, A. (2005). Qualitative and quantitative analyses of flower scent in *Silene latifolia* . Phytochemistry, 66, 203–213. 15652577 10.1016/j.phytochem.2004.12.002

[ece39920-bib-0022] Feng, H. H. , Wang, X. Y. , Luo, Y. B. , & Huang, S. Q. (2023). Floral scent emission is the highest at the second night of anthesis in *Lonicera japonica* (Caprifoliaceae). Journal of Systematics and Evolution. 10.1111/jse.12916

[ece39920-bib-0023] Fenster, C. B. , Armbruster, W. S. , Wilson, P. , Dudash, M. R. , & Thomson, J. D. (2004). Pollination syndromes and floral specialization. Annual Review of Ecology Evolution and Systematics, 35, 375–403.

[ece39920-bib-0024] Ferrer, M. M. , Good‐Avila, S. V. , Montaña, C. , Domínguez, C. A. , & Eguiarte, L. E. (2009). Effect of variation in self‐incompatibility on pollen limitation and inbreeding depression in *Flourensia cernua* (Asteraceae) scrubs of contrasting density. Annals of Botany, 103, 1077–1089. 19218580 10.1093/aob/mcp033 PMC2707912

[ece39920-bib-0025] Galetto, L. , Araujo, F. P. , Grilli, G. , Amarilla, L. D. , Torres, C. , & Sazima, M. (2018). Flower trade–offs derived from nectar investment in female reproduction of two *nicotiana* species (Solanaceae). Acta Botanica Brasilica, 32, 473–478.

[ece39920-bib-0027] Gaskett, A. C. , Conti, E. , & Schiestl, F. P. (2005). Floral odour variation in two heterostylous species of *primula* . Journal of Chemical Ecology, 31, 1223–1228. 16124243 10.1007/s10886-005-5351-9

[ece39920-bib-0028] Goodwin, R. M. , Cox, H. M. , Taylor, M. A. , Evans, L. , & Mcbrydie, H. (2011). Number of honey bee visits required to fully pollinate white clover (*Trifolium repens*) seed crops in Canterbury, New Zealand. New Zealand Journal of Crop and Horticultural Science, 39, 7–19.

[ece39920-bib-0029] Hagler, J. R. , & Buchmann, H. S. L. (1993). Honey bee (Hymenoptera: Apidae) foraging responses to phenolic–rich nectars. Journal of the Kansas Entomological Society., 66, 223–230.

[ece39920-bib-0105] Harder, L. D. , & Johnson, S. D. (2009). Darwin's beautiful contrivances: Evolutionary and functional evidence for floral adaptation. New Phytologist, 183, 530–545. 19552694 10.1111/j.1469-8137.2009.02914.x

[ece39920-bib-0030] Hoballah, M. E. , Stuurman, J. , Turlings, T. C. J. , Guerin, P. M. , Connétable, S. , & Kuhlemeier, C. (2005). The composition and timing of flower odour emission by wild *Petunia axillaris* coincide with the antennal perception and nocturnal activity of the pollinator *Manduca sexta* . Planta, 222, 141–150. 15891900 10.1007/s00425-005-1506-8

[ece39920-bib-0106] Hodges, S. A. (1995). The influence of nectar production on hawkmoth behavior, self pollination, and seed production in *Mirabilis multiflora* (Nyctaginaceae). American Journal of Botany, 82, 197–204.

[ece39920-bib-0032] Jarriault, D. , Barrozo, R. B. , Pinto, C. J. D. C. , Greiner, B. , Marie‐Cécile, D. , Masante‐Roca, I. , Gramsbergen, J. B. , Anton, S. , & Gadenne, C. (2009). Age‐dependent plasticity of sex pheromone response in the moth, *Agrotis ipsilon*: Combined effects of octopamine and juvenile hormone. Hormones and Behavior, 56, 185–191. 19409391 10.1016/j.yhbeh.2009.04.005

[ece39920-bib-0033] Johnson, B. O. , Golonka, A. M. , Blackwell, A. , Vazquez, I. , & Wolfram, N. (2019). Floral scent variation in the heterostylous species *Gelsemium sempervirens* . Molecules, 24, 2818. 31382381 10.3390/molecules24152818 PMC6695955

[ece39920-bib-0034] Johnson, S. D. , Balducci, M. G. , & Shuttleworth, A. (2019). Hawkmoth pollination of the orchid *Habenaria clavata*: Mechanical wing guides, floral scent and electroantennography. Biological Journal of the Linnean Society, 129, 1–14.

[ece39920-bib-0035] Jürgens, A. (2004). Nectar sugar composition and floral scent compounds of diurnal and nocturnal *Conophytum* species (Aizoaceae). South African Journal of Botany, 70, 191–205.

[ece39920-bib-0037] Kaiser, R. , & Tollsten, L. (1995). An introduction to the scent of cacti. Flavour and Fragrance Journal, 10, 153–164.

[ece39920-bib-0038] Keller, B. , Thomson, J. D. , & Conti, E. (2014). Heterostyly promotes disassortative pollination and reduces sexual interference in Darwin's primroses: Evidence from experimental studies. Functional Ecology, 28, 1413–1425.

[ece39920-bib-0039] Knudsen, J. T. , & Lars, T. (1993). Trends in floral scent chemistry in pollination syndromes: Floral scent composition in moth–pollinated taxa. Botanical Journal of the Linnean Society, 113, 263–284.

[ece39920-bib-0040] Kristina, F. , Anderson, K. M. , Rebecca, A. , Foster, M. C. , Foster, C. E. , Vik, D. , Vitt, P. , & Harris, M. O. (2015). Nectar robbery and thievery in the hawk moth (lepidoptera: Sphingidae)–pollinated western prairie fringed orchid *Platanthera praeclara* . Annals of the Entomological Society of America, 108, 1001–1013.

[ece39920-bib-0107] Leege, L. M. , & Wolfe, L. M. (2002). Do floral herbivores respond to variation in flower characteristics in *Gelsemium sempervirens* (Loganiaceae), a distylous vine? American Journal of Botany, 89, 1270–1274. 21665728 10.3732/ajb.89.8.1270

[ece39920-bib-0041] Leiss, K. A. , & Klinkhamer, P. (2005). Spatial distribution of nectar production in a natural *Echium vulgare* population: Implications for pollinator behaviour. Basic and Applied Ecology, 6, 317–324.

[ece39920-bib-0042] Li, Y. , Ma, H. , Wan, Y. , Li, T. , Liu, X. , Sun, Z. , & Li, Z. (2016). Volatile organic compounds emissions from *Luculia pinceana* flower and its changes at different stages of flower development. Molecules, 21, 531. 27110758 10.3390/molecules21040531 PMC6273779

[ece39920-bib-0043] Li, Y. , Wan, Y. , Sun, Z. , Li, T. , Liu, X. , Ma, H. , Liu, X. , He, R. , Ma, Y. , & Li, Z. (2017). Floral scent chemistry of *Luculia yunnanensis* (Rubiaceae), a species endemic to China with sweetly fragrant flowers. Molecules, 22, 879. 28587077 10.3390/molecules22060879 PMC6152718

[ece39920-bib-0044] Liu, C. Q. , & Huang, S. Q. (2013). Floral divergence, pollinator partitioning and the spatiotemporal pattern of plant–pollinator interactions in three sympatric *Adenophora* species. Oecologia, 173, 1411–1423. 23824141 10.1007/s00442-013-2723-7

[ece39920-bib-0045] Lloyd, D. G. , & Webb, C. J. (1992). The selection of heterostyly. In S. C. H. Barrett (Ed.), Evolution and function of heterostyly (pp. 179–207). Springer‐Verlag.

[ece39920-bib-0046] Lunau, K. , Konzmann, S. , Winter, L. , Kamphausen, V. , & Ren, Z. X. (2017). Pollen and stamen mimicry: The alpine flora as a case study. Arthropod‐Plant Interactions, 11, 427–447.

[ece39920-bib-0047] Manning, J. C. , & Snijman, D. (2002). Hawkmoth‐pollination in *Crinum variabile* (Amaryllidaceae) and the biogeography of sphingophily in southern African Amaryllidaceae. South African Journal Botany, 68, 212–216.

[ece39920-bib-0048] Matias, R. , Pérez‐Barrales, R. , & Consolaro, H. (2020). Patterns of variation in distylous traits and reproductive consequences in *Erythroxylum* species and populations. American Journal of Botany, 107, 910–922. 32462680 10.1002/ajb2.1478

[ece39920-bib-0049] McMullen, C. K. (2012). Pollination of the heterostylous Galápagos native, *Cordia lutea* (Boraginaceae). Plant Systematics and Evolution, 298, 569–579.

[ece39920-bib-0050] Mitchell, R. J. (2004). Heritability of nectar traits: Why do we know so little? Ecology, 85, 1527–1533.

[ece39920-bib-0051] Miyake, T. , & Yahara, T. (1998). Why does the flower of *Lonicera japonica* open at dusk? Canadian Journal of Botany, 76, 1806–1811.

[ece39920-bib-0052] Miyake, T. , Yamaoka, R. , & Yahara, T. (1998). Floral scents of hawkmoth–pollinated flowers in Japan. Journal of Medicinal Plants Research, 111, 199–205.

[ece39920-bib-0053] Nicolson, S. W. , Nepi, M. , & Pacini, E. (2007). Nectaries and nectar. Springer.

[ece39920-bib-0055] Pacini, E. , Nepi, M. , & Vesprini, J. L. (2003). Nectar biodiversity: A short review. Plant Systematics Evolution, 238, 7–21.

[ece39920-bib-0108] Pellmyr, O. (1986). Three pollination morphs in *Cimicifuga simplex*; incipient speciation due to inferiority incompetition. Oecologia, 68, 304–307. 28310144 10.1007/BF00384804

[ece39920-bib-0109] Pellmyr, O. , & Thien, L. B. (1986). Insect reproduction and floral fragrances: Keys to the evolution of the angiosperms? Taxon, 35, 76–85.

[ece39920-bib-0057] Potascheff, C.d. M. , de Brito, V. L. G. , Galetto, L. , Sebbenn, A. M. , & Oliveira, P. E. (2020). Nectar features, diurnal and nocturnal pollinators, and male fitness in *Qualea grandiflora* (Vochysiaceae). Plant Systematics and Evolution, 306, 1–12.

[ece39920-bib-0058] Raguso, R. A. (2008). Wake up and smell the roses: The ecology and evolution of floral scent. Annual Review of Ecology Evolution and Systematics, 39, 549–569.

[ece39920-bib-0059] Raguso, R. A. , Levin, R. A. , Foose, S. E. , Holmberg, M. W. , & Mcdade, L. A. (2003). Fragrance chemistry, nocturnal rhythms and pollination "syndromes" in *nicotiana* . Phytochemistry, 63, 265–284. 12737977 10.1016/s0031-9422(03)00113-4

[ece39920-bib-0060] Raguso, R. A. , Light, D. M. , & Pickersky, E. (1996). Electroantennogram responses of *Hyles lineata* (Sphingidae: Lepidoptera) to volatile compounds from *Clarkia breweri* (Onagraceae) and other moth–pollinated flowers. Journal of Chemical Ecology, 22, 1735–1766. 24227106 10.1007/BF02028502

[ece39920-bib-0061] Riffell, J. A. , Alarcón, R. , Abrell, L. , Davidowitz, G. , Bronstein, J. L. , & Hildebrand, J. G. (2008). Behavioral consequences of innate preferences and olfactory learning in hawkmoth–flower interactions. Proceedings of National Academy Sciences USA, 105, 3404–3409. 10.1073/pnas.0709811105 PMC2265144 18305169

[ece39920-bib-0062] Rio, C. M. D. , Baker, H. G. , & Baker, I. (1992). Ecological and evolutionary implications of digestive processes: Bird preferences and the sugar constituents of floral nectar and fruit pulp. Experientia, 48, 544–551.

[ece39920-bib-0063] Ruiz‐Zapata, T. , & Arroyo, M. T. K. (1978). Plant reproductive ecology of a secondary deciduous tropical forest in Venezuela. Biotropica, 10, 221–230.

[ece39920-bib-0064] Schlumpberger, B. O. , Cocucci, A. A. , Moré, M. , Sérsic, A. N. , & Raguso, R. A. (2009). Extreme variation in floral characters and its consequences for pollinator attraction among populations of an Andean cactus. Annals of Botany, 103, 1489–1500. 19342397 10.1093/aob/mcp075 PMC2701769

[ece39920-bib-0065] Schlumpberger, B. O. , & Raguso, R. A. (2008). Geographic variation in floral scent of *Echinopsis ancistrophora* (Cactaceae); evidence for constraints on hawkmoth attraction. Oikos, 117, 801–814.

[ece39920-bib-0066] Schondube, J. E. , & Rio, C. M. D. (2004). Sugar and protein digestion in flower piercers and hummingbirds: A comparative test of adaptive convergence. Journal of Comparative Physiology B, 174, 263–273. 10.1007/s00360-003-0411-3 14758501

[ece39920-bib-0067] Sun, S. G. , Huang, Z. H. , Chen, Z. B. , & Huang, S. Q. (2017). Nectar properties and the role of sunbirds as pollinators of the golden–flowered tea (*Camellia petelotii*). American Journal Botany, 104, 468–476. 10.3732/ajb.1600428 28298377

[ece39920-bib-0068] Terry, L. I. , Moore, C. J. , Roemer, R. B. , Brookes, D. R. , & Walter, G. H. (2021). Unique chemistry associated with diversification in a tightly coupled cycad‐thrips obligate pollination mutualism. Phytochemistry, 186, 112715. 33721794 10.1016/j.phytochem.2021.112715

[ece39920-bib-0069] Vandelook, F. , Janssens, S. B. , Gijbels, P. , Fischer, E. , & Abrahamczyk, S. (2019). Nectar traits differ between pollination syndromes in Balsaminaceae. Annals of Botany, 124, 1–11. 31120478 10.1093/aob/mcz072 PMC6758581

[ece39920-bib-0070] Wan, X. L. , Li, J. H. , Lao, C. , & Du, Y. J. (2015). Antennal lobe neurons of *Spodoptera litura* (lepidoptera: Noctuidae) and their responses to plant odours and sex pheromones. Acta Entomologica Sinica, 58, 223–236 (In Chinese with English abstract).

[ece39920-bib-0071] Wang, X. Y. , Hu, D. Y. , Chen, Y. , Xiang, M. D. , Tang, H. Q. , Yi, Y. , & Tang, X. X. (2022). Ancillary polymorphic floral traits between two morphs adaptive to hawkmoth pollination in distylous plant *Tirpitzia sinensis* (Linaceae). BMC Plant Biology, 22, 1–11. 35655126 10.1186/s12870-022-03659-w PMC9164504

[ece39920-bib-0073] Wiesenborn, W. D. , & Baker, T. C. (1990). Upwind flight to cotton flowers by *Pectinophora gossypiella* (lepidoptera: Gelechiidae). Environmental Entomology, 19, 490–493.

[ece39920-bib-0074] Willmer, P. (2011). Pollination and floral ecology. Princeton University Press.

[ece39920-bib-0075] Wolfe, L. M. , Massinga, P. H. , & Johnson, S. D. (2009). A quantitative evaluation of the distylous syndrome in *Sebaea grandis* (Gentianaceae). South African Journal of Botany, 75, 785–790.

[ece39920-bib-0076] Wu, L. Y. , Chang, F. F. , Liu, S. J. , Armbruster, W. S. , & Huang, S. Q. (2018). Heterostyly promotes compatible pollination in buckwheats: Comparisons of intraflower, intraplant, and interplant pollen flow in distylous and homostylous *Fagopyrum* . American Journal of Botany, 105, 108–116. 29532921 10.1002/ajb2.1013

[ece39920-bib-0077] Wykes, G. R. (1952). An investigation of the sugars presents in the nectar of flowers of various species. New Phytologist, 51, 210–215.

[ece39920-bib-0078] Zheng, Y. Y. , Huang, R. M. , Chen, M. Q. , & Su, D. S. (2020). Analysis of natural floral components of golden pomelo by solid phase microextraction combined with gas chromatography‐mass spectrometry. Journal of Food Safety and Quality, 11, 4602–4607 (In Chinese with English abstract).

