# Supplementary material for: Features of floral odor and nectar in the distylous Luculia pinceana (Rubiaceae) promote compatible pollination by hawkmoths
Source: Ecol Evol. 2023 Mar 21;13(3):e9920. doi: 10.1002/ece3.9920 (PMC10030271; doi:10.1002/ece3.9920)
Supplement: Supplementary file 1 — Table S1 [file ECE3-13-e9920-s001.docx]

Table S1 The plots where pollination observation was conducted and the pollination observation date, number of sessions and time of each session at each plot in 2019 (black words ) and 2020 (blue words) at Laoshan Provincial Nature Reserve.

| Sites | | Location | | | Pollinator observation date / No. of sessions / time of each session in 2019 (black word) |
| --- | --- | --- | --- | --- | --- |
|  | |  | |  | and 2020 (blue word) |
| 1 | Alitudes (m) | | 1762.73 ± 4.23 | | June 22 / 7 / 0700-0730, 0730-0800, 0800-0830, 1100-1130, 1230-1300, 1300-1330, 1650-1720 |
|  | Longitudes | | 104°49′52″ | | June 26 / 7 / 1400-1430, 1430-1500, 1900-1930, 1930-2000, 2010-2040, 2100-2130, 2130-2200 |
|  | Latitudes | | 23°35′16″ | | July 14 / 6 / 0700-0730, 0800-0830, 1830-1900, 1930-2000, 2030-2100, 2130-2200 |
|  |  | |  | | July 16 / 6 / 1030-1100, 1200-1230, 1700-1730, 1900-1930, 2100-2130, 2130-2200 |
|  |  | |  | | July 17 / 4 / 1400-1430, 1500-1530, 2030-2100, 2130-2200 |
| 2 | Alitudes (m) | | 1567.63 ± 4.33 | | June 27 / 6 / 0830-0900, 0930-1000, 1200-1230, 1830-1900, 1900-1930, 1930-2000 |
|  | Longitudes | | 104°29′49″ | | June 28 / 4 / 1700-1730, 2000-2030, 2030-2100, 2130-2200 |
|  | Latitudes | | 23°24′50″ | | July 18 / 5 / 0700-0730, 0900-0930, 1800-1830, 2000-2030, 2030-2100 |
|  |  | |  | | July 23 / 6 / 1200-1230, 1300-1330, 1700-1730, 1930-2000, 2030-2100, 2130-2200 |
|  |  | |  | | July 24 / 4 / 1530-1600, 1900-1930, 2000-2030, 2130-2200 |
| 3 | Alitudes (m) | | 1423.82 ± 2.89 | | June 29 / 5 / 0700-0730, 0900-0930, 1100-1130, 2000-2030, 2030-2100 |
|  | Longitudes | | 104°20′12″ | | June 30 / 5 / 1200-1230, 1500-1530, 1700-1730, 2040-2110, 2130-2200 |
|  | Latitudes | | 23°34′52″ | | July 26 / 2 / 0700-0730, 0800-0830 |
|  |  | |  | | July 27 / 5 / 1100-1130, 1230- 1300, 1830-1900, 1930-2000, 2130-2200 |
| 4 | Alitudes (m) | | 1362.21 ± 3.80 | | July 1 / 7 / 0900-0930, 0930-1000, 1315-1345, 1345-1415, 1515-1545, 2020-2050, 2130-2200 |
|  | Longitudes | | 104°30′43″ | | August 1 / 5 / 0700-0730, 1000-1030, 1830-1900, 1930-2000, 2030-2100 |
|  | Latitudes | | 23°20′09″ | | August 4 / 2/ 1400-1430, 1500-1530 |
| 5 | Alitudes (m) | | 1249.31 ± 3.47 | | July 3 / 3 / 1430-1500, 1500-1530, 1530-1600 |
|  | Longitudes | | 104°25′05″ | | August 5 / 5 / 0700-0730, 1000-1030, 1830-1900, 1930-2000, 2030-2100 |
|  | Latitudes | | 23°35′17″ | | August 9 / 2 / 1200-1230, 1400-1430 |
